# Association between air pollution and emergency department visits for unintentional injuries in four cities of Shandong Province, China

**DOI:** 10.3389/fpubh.2025.1710542

**Published:** 2025-12-09

**Authors:** Yanli Dong, Yanxin Gao, Yizhi Liu, Dongyue He, Huan Li, Huiying Ding, Xuting Yang, Yanmeng Kang, Xuena Liu

**Affiliations:** 1 School of Public Health, Shandong First Medical University and Shandong Academy of Medical Sciences, Jinan, Shandong Province, China; 2 Shandong Provincial Center for Disease Control and Prevention, Jinan, Shandong Province, China; 3 The First Affiliated Hospital of Shandong First Medical University, Jinan, Shandong Province, China

**Keywords:** unintentional injuries, air pollutants, dynamic factor model, distributed lag nonlinear model, emergency department visits

## Abstract

**Objective:**

To study the relationship between emergency department visits for unintentional injuries and air pollutants in four cities in Shandong Province, China (2019–2022).

**Methods:**

From 2019 to 2022, the data on air pollutants and unintentional injury emergency department visits were extracted from four cities in Shandong Province (Dezhou, Jinan, Zibo, and Heze). We used the dynamic factor model (DFM) to determine the trend of emergency visits and the distributed lag nonlinear model (DLNM) to analyze the lag effect of air pollutants on these visits. We also conducted a meta-analysis to summarize the impact estimates of air pollutants on emergency visits.

**Results:**

The dynamic factor model showed that from 2019 to 2022, the number of emergency visits for unintentional injuries in the four cities in Shandong Province was cyclical, with the highest number of emergency visits in summer every year. PM_2.5_, PM_10_, CO, SO_2_, NO_2_, and O_3_ exhibited single-day lag effects on emergency visits for unintentional injuries, with the highest risk observed at Lag4, Lag5, Lag4, Lag3, Lag1, and Lag3, respectively. The corresponding relative risks (*RRs*) and 95% confidence intervals (*CIs*) were 1.014(1.007, 1.021), 1.015(1.006, 1.023), 1.085(1.038, 1.134), 1.036(1.013, 1.060), 1.121(1.069, 1.175), and 1.084(1.052, 1.116). Additionally, PM_2.5_, PM_10_, CO, SO_2_, NO_2_, and O_3_ demonstrated cumulative lag effects, with the highest risk observed at Lag07, Lag07, Lag06, Lag05, Lag06, and Lag05, respectively. The *RRs (95% CIs)* were 1.058(1.033, 1.083), 1.037(1.003, 1.072), 1.468(1.244, 1.733), 1.099(1.039, 1.161), 1.221(1.097, 1.359), and 1.227(1.138, 1.323).

**Conclusion:**

Emergency visits for unintentional injuries in the four cities exhibited an annual cyclical pattern, with the peak occurring in summer. Exposure to high concentrations of air pollutants increased the risk of unintentional injuries, with distinct effects observed across different pollutants, each exhibiting specific single-day and cumulative lag effects.

## 1 Introduction

Unintentional injuries pose a significant challenge to the global public health pattern, bringing a major burden to individuals, families and society as a whole. In particular, the total number of patients with unintentional injuries in emergency departments around the world is increasing significantly. This increase puts pressure on already limited resources and places an additional burden on the efficiency of emergency department systems and health care systems for emergency response, treatment and resource use. Unintentional injury is defined as an injury caused by unforeseen or inevitable external events, which is characterized by sudden and unexpected occurrence. This distinguishes it from diseases with specific etiologies (1). Unintentional injuries include a wide range of environmental events, such as poisoning, suffocation, drowning, falls, motor vehicle collisions, burns, and scalds (2), and most of the events are preventable (3). Emergency department visit data are a key indicator for the real-time reassessment of regional emergency service capacity and population health. Compared with hospitalization or mortality data, emergency visits can describe the phenomena affecting acute health outcomes more directly, accurately and specifically (4). Unintentional injuries are a considerable part of the total number of nursing patients related to the emergency department. In recent years, the focus of public health has shifted to issues related to the decline in air quality. Previous studies have identified the association between injuries and air pollutants in the environment, with higher levels being linked to an increased risk of injury (5). There has been less quantitative empirical analysis of the relationship between air pollutants and unintentional injuries. In order to fill this gap, this study was completed in four cities in Shandong Province to examine the time trend of emergency visits related to unintentional injuries, as well as to quantify the relationship exposure-response and lag effects between air pollutants and emergency visits. The findings not only provide scientific evidence for injury prevention measures in Shandong Province, but their methodology and core conclusions also offer a valuable reference for public health decision-making in regions with similar climates.

## 2 Materials and methods

### 2.1 Study area

This study was conducted in four inland cities of Shandong Province, China: Dezhou, Jinan, Zibo, and Heze. All four cities are located in inland areas (non-coastal areas) of Shandong Province and share similar climatic conditions, economic levels, and population sizes. This selection comprehensively represents the impact of air pollutants on emergency department visits for unintentional injuries in inland areas of Shandong Province, particularly the northwestern region.

### 2.2 Data sources

#### 2.2.1 Disease data

Data from all emergency medical services (EMS) cases (via 120 or 999 calls) within urban and county areas of four cities between 2019 and 2022 were collected, primarily including general information, date of admission, chief complaint at admission, location of emergency call, initial diagnosis, and ICD codes. Unintentional injuries primarily encompassed traffic accidents, falls, drowning, poisoning, burns, and mechanical asphyxia. When screening subjects for this study, we selectively included unintentional injury cases clearly corresponding to ICD-10 codes V01-V99, W00-X59, and Y40-Y84, based on the International Classification of Diseases, Tenth Revision (ICD-10) coding system, and combined with patients’ chief complaints and clinical diagnoses. Additionally, code S00-T98, which refers to “Injury, poisoning, and certain other consequences of external causes” include some unintentional injury events. Subsequently, we manually reviewed the original medical records with a focus on the “cause of injury” description, retaining only those containing clear unintentional injury keywords, such as “head injury due to a traffic accident,” and “fracture after an accidental fall.” By applying this dual-approach combining code ranges and keyword characteristics, we ensured that all included cases represented unintentional injury events, thereby enhancing the reliability of the research data (1).

#### 2.2.2 Air pollutant data

Air pollutant data were obtained from the China Air Quality Online Monitoring and Analysis Platform,^1^ including daily concentrations of particulate matter (PM_2.5_, PM_10_), CO, SO_2_, NO_2_, and O_3_. Missing data were imputed using multiple imputation methods.

### 2.2.3 Meteorological data

Our meteorological data source was the China Meteorological Data Sharing Service System.^2^ The meteorological data in our study included average temperature (°C) and relative humidity (%).

## 2.3 Statistical analysis

### 2.3.1 Dynamic factor model

The dynamic factor model (DFM) (6) was used to analyze unintentional injuries in the emergency department. The general structure of the model is as follows: Equations (1), (2), and (3):


(1) $$y_{t}=E_{i}\;f_{t}+H\;x_{t}+u_{t}$$

(2) $$f_{t}=K\;w_{t}+A_{1}\;f_{t-1}+A_{2}\;f_{t-2}+\ldots +A_{1-p}\;f_{t-p}+v_{t}$$

(3) $$u_{t}=C_{1}\;u_{t-1}+C_{2}\;u_{t-2}+\ldots +C_{t-q}\;u_{1-q}+\varepsilon_{t}$$

Here, *y_t_* represents the observed variables, which are the n time series data sets of the observation results. *f_t_* is a common factor vector that reflects the unobserved common trends in the n time series. *Eᵢ* is the factor loading matrix of the ith observation sequence, and *u_t_* signifies the idiosyncratic component, capturing random fluctuations specific to each time-series that are not directly associated with the common factors. Both *f_t_* and *u_t_* exhibit autoregressive structures of orders *p* and *q*, and *A_i_* and *C_i_* are their autoregressive parameter matrices. The disturbance vectors of the two autoregressive equations are *v_t_* and *ε_t_*, *x_t_* and *w_t_* are also the potential exogenous variable vectors considered in the equation, and *H_i_* and *K_i_* are their corresponding parameter matrices.

For the DFM construction in this study, a single common factor was extracted to analyze the observed variables, with the significance level *α* set at 0.05. The autoregressive orders for both common and idiosyncratic factors were determined through the Bayesian information criterion (*BIC*) estimation (6).

### 2.3.2 Distributed lag nonlinear model

The DLNM (7) was constructed using the “dlnm” package in *R* software (version 4.4.1) to assess the lag effects of air pollution on unintentional injuries. A quasi-Poisson regression model was chosen due to the characteristics of the emergency department data. Cross-basis functions were applied to all pollutants to generate the cross-basis matrices, in which natural cubic splines were used for the pollutant concentration dimension, while the lag dimension used splines with knots generated by the logknots function, internal knots were placed at the 10th, 50th, and 90th percentiles of the pollutant concentration distribution, with the maximum lag set at 7 days. The model also adjusted for confounders in the study: a cross-basis function was used to control for the immediate and lagged effects of temperature, where natural cubic splines were used as the basis function for the immediate effect of temperature, and splines with knots generated by the logknots function were used for the lagged effect of temperature. Additionally, natural spline functions were employed to control for relative humidity (*df* = 3) and long-term temporal trends (*df* = 7/year). Day-of-week effects, holiday effects, and the impact of the COVID-19 pandemic were included in the model as factor variables.

The model was formulated as follows: Equation (4):


(4) $$\begin{array}{l} \log[E(Y_{t)}]=\alpha +\mathrm{cb}.(\text{pollutan}t_{t}))+\mathrm{cb}.(\text{temperatur}e_{t})+\\ \mathrm{ns}(\text{humidit}y_{t},\mathrm{df}=3)+\mathrm{ns}(\text{time},\mathrm{df}=7/\text{year})+\\ \mathrm{as}.\text{factor}(\mathrm{dow})+\mathrm{as}.\text{factor}(\text{holiday})+\\ \mathrm{as}.\text{factor}(\text{case}) \end{array}$$

In the equation: *E(Y_t_)* represents the expected value of emergency visits due to unintentional injuries; *α* denotes the intercept term; *cb* represents the cross-term basis function; *ns* represents the natural cubic spline; *temperature_t_* denotes the daily average concentration of temperature; *humidity_t_* denotes the daily average concentration of humidity; *pollutant_t_* denotes the daily average concentration of air pollutants; *time* is the time series variable; *df* denotes the degrees of freedom; *dow* denotes the day-of-the-week effect; *holiday* denotes the holiday effect; *case* denotes the COVID-19 pandemic effect, which was included in the DLNM model as a binary variable (regional lockdown = 1, no regional lockdown = 0).

This model is expected to obtain association effect values for each air pollutant and the volume of emergency visits for unintentional injuries across the four cities. All effect values are presented as *RR* values, with the fifth percentile (P_5_) concentration of each of the six air pollutants serving as the baseline reference. Specifically, the *RR* value represents the relative risk change in the volume of emergency visits for unintentional injuries when the concentration of a specific pollutant exceeds its P_5_ concentration (7). Additionally, a maximum lag period of 7 days was set. The model assessed both the single-day lag effects (Lag0-Lag7) and cumulative lag effects (Lag01-Lag07) of air pollutants on emergency department visits for unintentional injuries.

### 2.3.3 Sensitivity analysis

In order to evaluate the robustness of the model, sensitivity analyses were carried out by changing the *df* of the time trend or adding other air pollutants, while the stability of the model was evaluated by assessing the changes in the values of the Akaike information criterion (*AIC*).

### 2.3.4 Meta analysis

Using Stata 18, a meta-analysis was carried out to quantitatively evaluate air pollution and emergency visits from four cities in Shandong Province, including single-day and cumulative lag effects.

## 3 Results

### 3.1 Baseline characteristics of emergency visits and air pollution data

#### 3.1.1 Emergency visit data

From 2019 to 2022, there were more than 619,003 emergency visits for unintentional injuries across four cities in Shandong Province. The number of emergency visits in each city was: Jinan, 167,011 cases, Zibo, 122,526 cases, Dezhou, 155,111 cases, Heze, 174,355 cases. The daily average of each city was 114, 83, 106, and 119, respectively (Table 1).

**Table 1:** Statistical description of average daily emergency visits for unintentional injury in four cities of Shandong Province, 2019–2022.

Variable	Mean (SD)	Min	P_25_	P_50_	P_75_	Max
Jinan	114(35)	19	92	112	135	227
Zibo	83(32)	17	69	89	106	166
Dezhou	106(26)	23	89	106	125	195
Heze	119(27)	21	103	120	138	203

**Figure 1:**
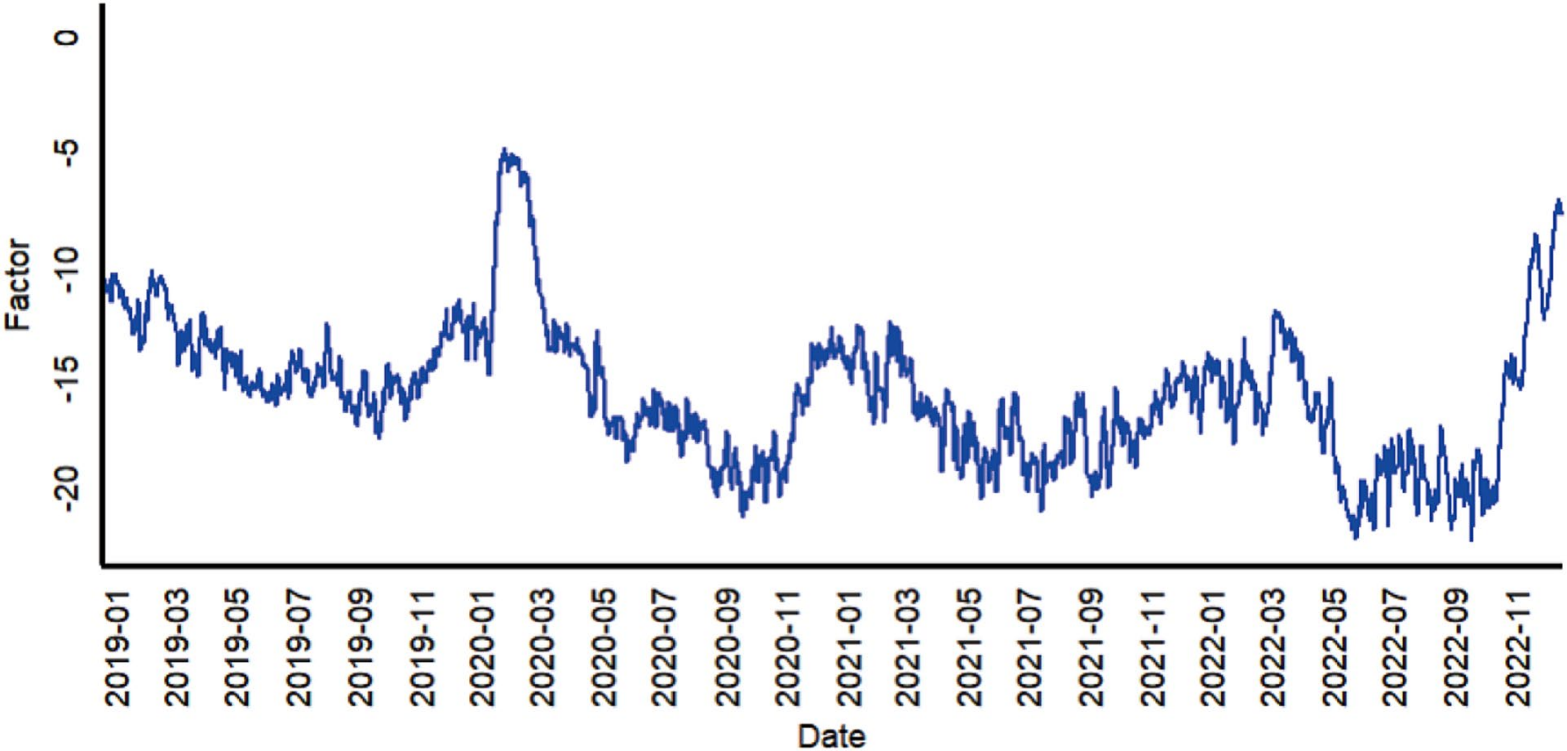
Estimated common factors for daily emergency department visits due to unintentional injuries in four cities of Shandong Province, 2019–2022.

This study applied the dynamic factor model (DFM), extracted the common factors related to unintentional injuries using data from the emergency departments in four cities, followed by an analysis of the time trend of emergency visits. During model construction, since the emergency visit data were a stationary time series, no differencing was applied. Based on the fitting and evaluation of the BIC, the autoregressive order of common factors was set to 2, and the autoregressive order of idiosyncratic factors was set to 1. Hypothesis tests were carried out on the dynamic factor model and parameters, indicating that the dynamic factor model was stable (*p <* 0.05). Following parameter estimation, common factor estimates were further obtained, as detailed in Table 2. Figure 1 reflects the trend of common factors for going to the emergency department due to unintentional injuries in each region.

**Table 2:** Parameter estimation and hypothesis testing results of the common factor model of unintentional injury emergency visits in four cities in Shandong Province, 2019–2022.

Variable	Lag	Ratio	Standard error	Statistic	*p*
Common factor	First-order lag	0.45	0.04	10.99	<0.001
	second-order lag	0.55	0.04	13.56	<0.001
Jinan special factor	First-order lag	0.71	0.02	31.48	<0.001
Zibo special factor	First-order lag	0.88	0.01	64.04	<0.001
Dezhou special factor	First-order lag	0.26	0.04	6.33	<0.001
Heze special factor	First-order lag	0.45	0.04	12.40	<0.001
Jinan	-	-6.87	0.39	−17.71	<0.001
Zibo	-	−5.16	0.34	−15.26	<0.001
Dezhou	-	−6.33	0.35	−17.85	<0.001
Heze	-	−7.05	0.39	−17.91	<0.001

The extracted common factors reflected fluctuation patterns in emergency visits after detrending. Analysis of the common factor estimates revealed an annual cyclical variation with a trend component, where troughs predominantly occurred in summer. It is thus inferred that the number of emergency department visits for unintentional injuries exhibits cyclical fluctuations, with peaks mainly concentrated in summer.

Based on the model parameter estimates in Table 2, the standardized sequence data for emergency department visits for unintentional injuries can be expressed using a set of equations in the following Equations 5–9 format:


(5) $$y_{\text{dezhou}}=-6.33f_{t}+0.26u_{t-1}$$

(6) $$y_{\text{jinan}}=-6.87f_{t}+0.71u_{t-1}$$

(7) $$y_{\text{zibo}}=-5.16f_{t}+0.88u_{t-1}$$

(8) $$y_{\text{heze}}=-7.05f_{t}+0.45u_{t-1}$$

(9) $$\text{ft}=0.45\;f_{t-1}+0.55\;f_{t-2}$$

#### 3.1.2 Air pollutant data

The average concentrations of PM_2.5_, PM_10_, CO, SO_2_, NO_2_, and O_3_ in Jinan, Zibo, Dezhou, and Heze, in Shandong Province, from 2019 to 2022 are detailed in Table 3.

**Table 3:** Statistical description of air pollutant concentrations in four cities of Shandong Province, 2019–2022.

City	Air pollutant	Mean (SD)	Min	P25	P50	P75	Max
Dezhou	NO_2_	28.82(15.44)	4.0	17.0	24.0	38.0	88.0
	SO_2_	11.61(6.11)	2.0	8.0	10.0	14.0	55.0
	O_3_	111.83(51.74)	7.0	70.0	106.0	148.0	288.0
	PM_2.5_	46.01(39.13)	0.0	22.0	35.0	55.0	310.0
	PM_10_	86.35(55.40)	0.0	48.0	74.0	111.0	419.0
	CO	0.83 (2.87)	0.2	0.6	0.7	0.9	110.0
Jinan	NO_2_	36.06(16.79)	7.0	23.0	34.0	46.0	110.0
	SO_2_	12.47(6.25)	4.0	8.0	11.0	15.0	52.0
	O_3_	111.74(54.21)	9.0	67.0	106.0	151.5	284.0
	PM_2.5_	44.66(33.11)	0.0	24.0	36.0	55.0	278.0
	PM_10_	86.35(50.34)	0.0	52.5	76.0	108.0	386.0
	CO	0.81 (0.32)	0.3	0.6	0.7	0.9	3.2
Zibo	NO_2_	36.83(15.27)	8.0	25.0	34.0	47.0	103.0
	SO_2_	16.89(8.36)	4.0	11.0	15.0	21.0	62.0
	O_3_	113.75(55.11)	6.0	69.0	106.0	154.0	284.0
	PM_2.5_	49.92(35.18)	0.0	28.0	41.0	61.0	262.0
	PM_10_	86.04(50.01)	0.0	54.0	76.0	108.0	359.0
	CO	0.91(0.43)	0.2	0.6	0.8	1.1	4.4
Heze	NO_2_	27.78(13.31)	6.0	18.0	24.0	36.0	80.0
	SO_2_	11.53(7.59)	3.0	7.0	10.0	14.0	68.0
	O_3_	109.10(48.14)	5.0	69.0	105.0	144.0	268.0
	PM_2.5_	51.66(39.54)	0.0	26.0	39.0	64.0	285.0
	PM_10_	97.74(57.48)	0.0	60.0	88.0	125.5	414.0
	CO	0.69 (0.29)	0.1	0.5	0.6	0.8	2.5

^*^NO_2_(μg/m^3^), SO_2_(μg/m^3^), O_3_(μg/m^3^), PM_2.5_(μg/m^3^), PM_10_(μg/m^3^), CO(mg/m^3^).

### 3.2 Impact of air pollution on emergency visits for unintentional injuries across cities

**Figure 2:**
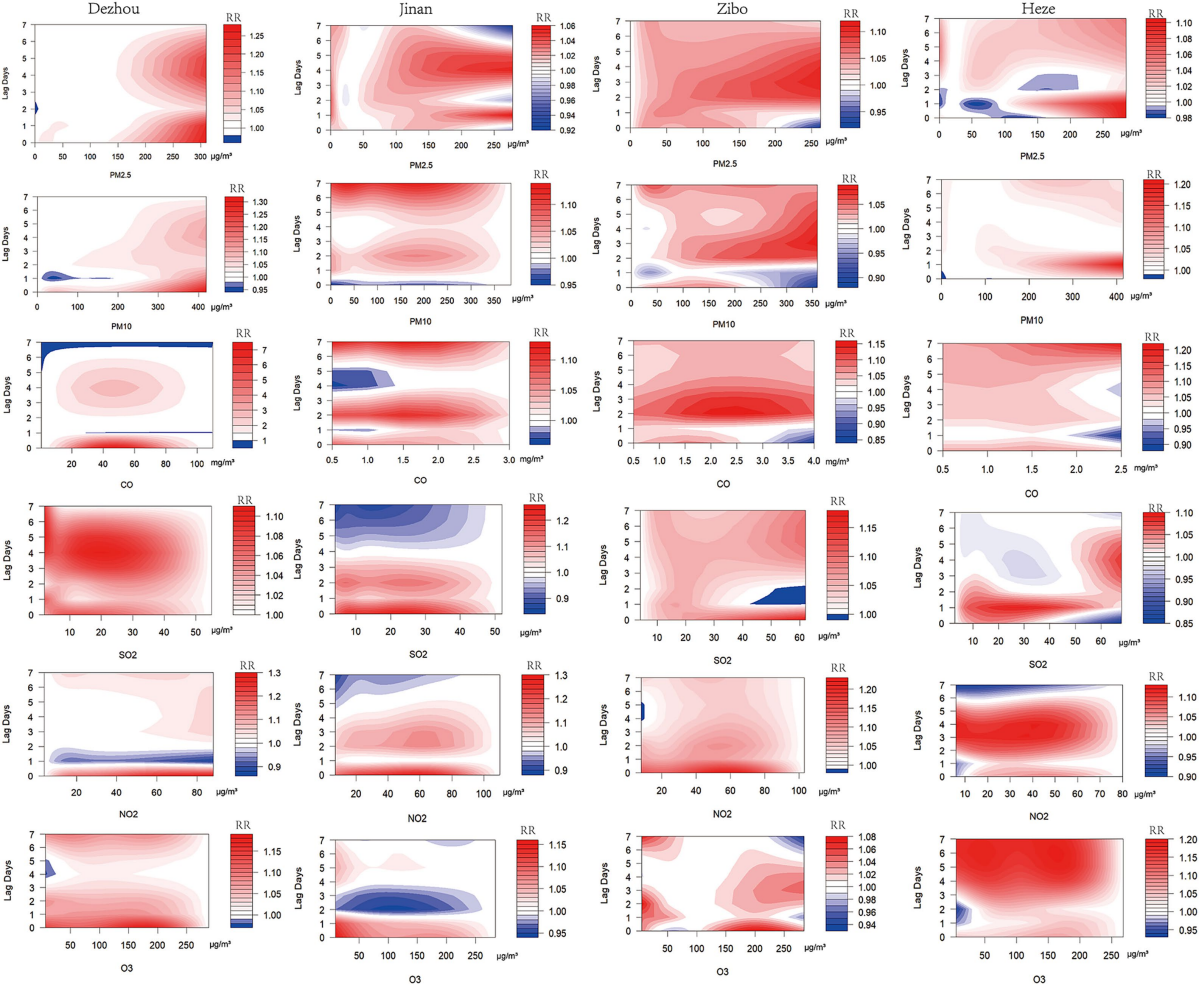
Contour plots of the *RR* values across four cities as a function of air pollutants and lag time.

**Figure 3:**
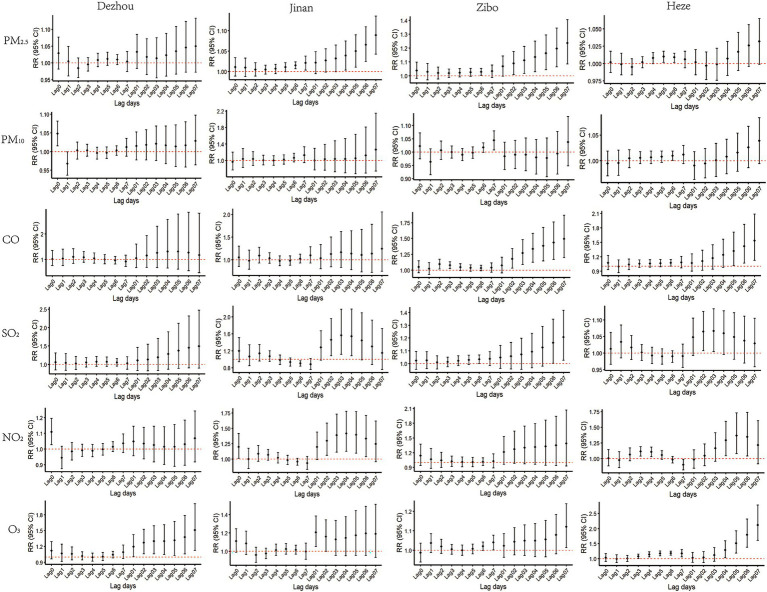
Single-day and cumulative lag effects of air pollutants on emergency department visits for unintentional injuries across cities.

The association between air pollutants and emergency department visits for unintentional injuries was assessed using a DLNM. As shown in the contour plots (Figure 2), the hazardous effect of PM_10_ in Jinan began to appear after lag day 1 (Lag1), while the other pollutants exhibited significant hazardous effects on the same day of exposure (Lag0), along with noticeable lagged effects. The single-day and cumulative lag effects of air pollutants on emergency visits are presented in Table 4 and Figure 3.

**Table 4:** Quantitative analysis of associations between air pollutants and emergency department visits for unintentional injuries across four cities.

Air pollutant	City	Single-day lag effect		Cumulative lag effect	
PM_2.5_	Dezhou	1.029(0.982,1.077)	Lag0	1.050(0.973,1.133)	Lag07
	Jinan	1.021(1.004,1.038)	Lag7	1.089(1.043,1.136)	Lag07
	Zibo	1.029(1.004,1.055)	Lag6	1.236(1.087,1.406)	Lag07
	Heze	1.010(1.002,1.018)	Lag5	1.032(0.999,1.065)	Lag07
PM_10_	Dezhou	1.048(1.016,1.082)	Lag0	1.029(0.965,1.098)	Lag07
	Jinan	1.126(0.951,1.332)	Lag7	1.264(0.742,2.154)	Lag07
	Zibo	1.044(1.008,1.080)	Lag7	1.037(0.948,1.134)	Lag07
	Heze	1.010(1.001,1.019)	Lag6	1.039(0.995,1.084)	Lag07
CO	Dezhou	1.100(0.848,1.425)	Lag2	1.315(0.621,2.783)	Lag05
	Jinan	1.096(0.929,1.291)	Lag7	1.243(0.750,2.061)	Lag07
	Zibo	1.095(1.021,1.174)	Lag2	1.496(1.198,1.869)	Lag07
	Heze	1.074(1.008,1.144)	Lag6	1.536(1.127,2.093)	Lag07
SO_2_	Dezhou	1.074(0.949,1.217)	Lag4	1.495(0.902,2.477)	Lag07
	Jinan	1.198(0.948,1.515)	Lag0	1.565(1.122,2.183)	Lag03
	Zibo	1.033(1.001,1.067)	Lag6	1.207(1.026,1.419)	Lag07
	Heze	1.034(0.986,1.085)	Lag1	1.067(1.005,1.134)	Lag03
NO_2_	Dezhou	1.110(1.029,1.197)	Lag0	1.070(0.920,1.244)	Lag07
	Jinan	1.199(1.014,1.417)	Lag0	1.418(1.130,1.780)	Lag04
	Zibo	1.140(0.943,1.378)	Lag0	1.39(0.933,2.070)	Lag07
	Heze	1.114(1.040,1.193)	Lag3	1.371(1.083,1.735)	Lag05
O_3_	Dezhou	1.120(0.968,1.295)	Lag0	1.508(1.131,2.011)	Lag07
	Jinan	1.112(0.991,1.247)	Lag0	1.207(1.052,1.386)	Lag01
	Zibo	1.040(1.002,1.078)	Lag7	1.121(1.014,1.240)	Lag07
	Heze	1.185(1.119,1.255)	Lag6	2.118(1.611,2.784)	Lag07

#### 3.2.1 Quantitative relationship and lag effects of air pollutants on emergency department visits for unintentional injuries in four cities

##### 3.2.1.1 PM_2.5_

The highest-risk single-day lag periods for PM_2.5_ exposure on emergency department visits for unintentional injuries in Jinan, Zibo, and Heze occurred at Lag7, Lag6, and Lag5, with *RRs* and 95% *CIs* of 1.021(1.004, 1.038), 1.029(1.004, 1.055), and 1.010(1.002, 1.018), respectively. For cumulative lag effects, in Jinan and Zibo, the highest-risk lag periods occurred at Lag07, with *RRs* and 95% *CIs* of 1.089(1.043, 1.136) and 1.236(1.087, 1.406), respectively.

##### 3.2.1.2 PM_10_

The highest-risk single-day lag periods of PM_10_ on emergency department visits for unintentional injuries in Dezhou, Zibo, and Heze were Lag0, Lag7, and Lag6, respectively, with *RR* values and 95% *CIs* of 1.048(1.016, 1.082), 1.044(1.008, 1.080), and 1.010(1.001, 1.019), respectively.

##### 3.2.1.3 CO

The highest-risk single-day lag periods for the impact of CO on emergency department visits for unintentional injuries in Zibo and Heze were Lag2 and Lag6, respectively, with *RR* values and 95% *CIs* of 1.095(1.021, 1.174) and 1.074(1.008, 1.144). The highest-risk cumulative lag periods for Zibo and Heze were Lag07, respectively, with *RR* values and 95% *CIs* of 1.496(1.198, 1.869) and 1.536(1.127, 2.093).

##### 3.2.1.4 SO_2_

The highest-risk single-day lag periods of SO_2_ on emergency department visits for unintentional injuries were Lag6 in Zibo, with corresponding *RRs* and 95% *CIs* of 1.033(1.001, 1.067), respectively. For cumulative lag effects, the periods with the highest risk were Lag03 in Jinan, Lag07 in Zibo, and Lag03 in Heze, with *RRs* and 95% *CIs* of 1.565(1.122, 2.183), 1.207(1.026, 1.419), and 1.067(1.005, 1.134), respectively.

##### 3.2.1.5 NO_2_

The highest-risk single-day lag periods for NO_2_ on emergency department visits for unintentional injuries in Dezhou, Jinan and Heze were Lag0, Lag0 and Lag4, respectively, with *RR* values and 95% *CIs* of 1.110(1.029, 1.197), 1.199(1.014, 1.417), and 1.114(1.040, 1.193). The highest-risk cumulative lag periods in Jinan and Heze were Lag04 and Lag05, respectively, with *RR* values and 95% *CIs* of 1.418(1.130, 1.780) and 1.371(1.083, 1.735).

##### 3.2.1.6 O_3_

The highest-risk single-day lag periods for the impact of O_3_ on emergency department visits for unintentional injuries in Zibo and Heze were Lag7 and Lag6, respectively, with *RR* values and 95% *CIs* of 1.040(1.002, 1.078) and 1.185(1.119, 1.255). The highest-risk cumulative lag periods for Dezhou, Jinan, Zibo, and Heze were Lag07, Lag01, Lag07, and Lag07, respectively, with *RR* values and 95% *CIs* of 1.508(1.131, 2.011), 1.207(1.052, 1.386), 1.121(1.014, 1.240), 2.118(1.611, 2.784). Detailed results are presented in Table 4.

#### 3.2.2 Sensitivity analysis

To assess model stability, sensitivity analyses were performed by varying the *df* (6–10) for temporal trends or adding other pollutants. The results demonstrated that the AIC value did not increase substantially, indicating robust model stability. Detailed data are presented in Table 5.

**Table 5:** Model AIC values after altering the temporal degrees of freedom or adding pollutants.

City	Model	Variable	PM_2.5_	PM_10_	CO	SO_2_	NO_2_	O_3_
Dezhou	Vary the temporal *df*	*df* = 6/year	13,433	13,435	13,436	13,490	13,422	13,475
		*df* = 7/year	13,253	13,244	13,260	13,308	13,255	13,310
		*df* = 8/year	13,288	13,290	13,282	13,373	13,335	13,362
	Add other pollutants	+CO	13,276	13,299	-	-	-	-
		+SO_2_	13,297	13,291	13,286	-	-	-
		+NO_2_	13,207	13,229	13,254	13,249	-	-
		+ O_3_	13,293	13,274	13,294	13,342	13,265	-
Jinan	Vary the temporal *df*	*df* = 6/year	13,178	13,225	13,174	13,181	13,021	13,205
		*df* = 7/year	12,971	13,007	12,972	12,991	12,868	13,023
		*df* = 8/year	12,982	13,035	12,938	13,002	12,882	13,074
	Add other pollutants	+CO	12,977	13,009	-	-	-	-
		+SO_2_	12,970	12,997	12,953	-	-	-
		+NO_2_	12,831	12,861	12,789	12,851	-	-
		+ O_3_	13,009	13,033	12,987	13,007	12,884	-
Zibo	Vary the temporal *df*	*df* = 6/year	14,216	14,134	14,209	14,244	14,118	14,170
		*df* = 7/year	13,775	13,392	13,778	13,777	13,650	13,778
		*df* = 8/year	13,445	13,705	13,400	13,393	13,367	13,424
	Add other pollutants	+CO	13,832	13,768	-	-	-	-
		+SO_2_	13,790	13,752	13,814	-	-	-
		+NO_2_	13,637	13,638	13,618	13,697	-	-
		+ O_3_	13,788	13,737	13,800	13,815	13,693	-
Heze	Vary the temporal *df*	*df* = 6/year	14,567	14,558	14,531	14,548	14,388	14,476
		*df* = 7/year	14,376	14,356	14,381	14,380	14,275	14,239
		*df* = 8/year	14,303	14,290	14,273	14,307	14,195	14,162
	Add other pollutants	+CO	14,352	14,369	-	-	-	-
		+SO_2_	14,393	14,376	14,380	-	-	-
		+NO_2_	14,281	14,278	14,274	14,324	-	-
		+ O_3_	14,243	14,209	14,267	14,217	14,065	-

### 3.3 Comprehensive effects of air pollutants on emergency visits for unintentional injuries

**Figure 4:**
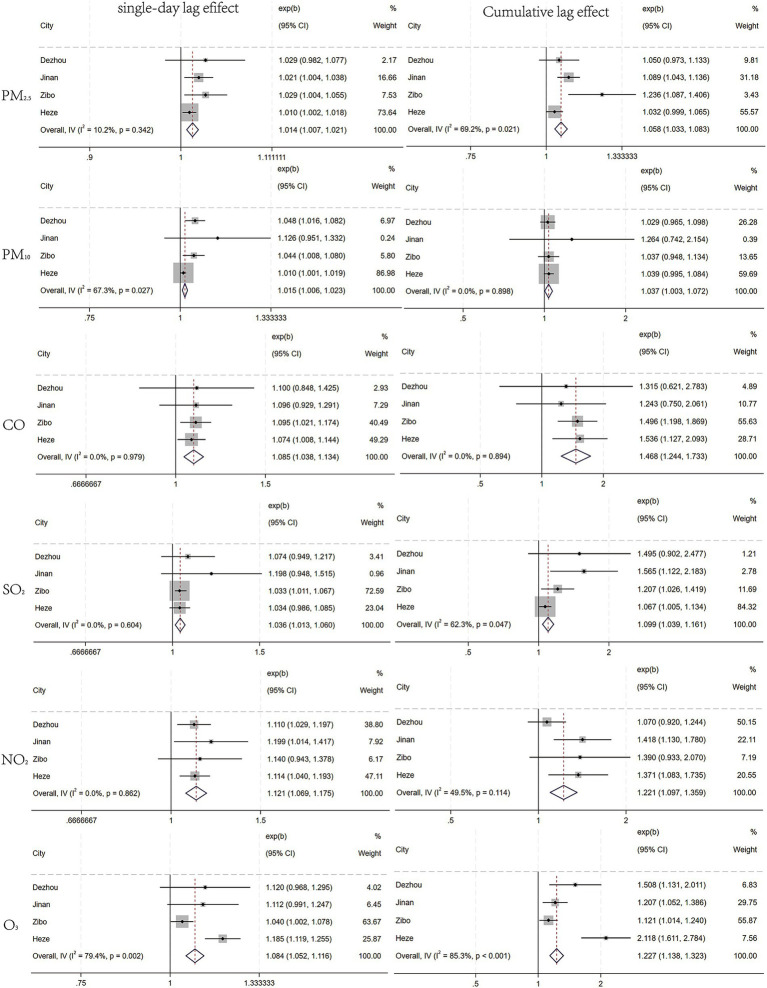
Pooled effect estimates of air pollution on emergency department visits for unintentional injuries across four cities.

The meta-analysis integrating data from the four cities revealed distinct lag patterns in the association between air pollutants and emergency visits for unintentional injuries. For PM_2.5_, the single-day lag and cumulative lag periods with the highest risk were identified as Lag4 and Lag07, with pooled *RRs* of 1.014(1.007, 1.021) and 1.058(1.033, 1.083), respectively. For PM_10_, the single-day lag and cumulative lag periods with the highest risk were Lag5 and Lag07, with pooled *RRs* of 1.015(1.006, 1.023) and 1.037(1.003, 1.072). For CO, the single-day lag and cumulative lag periods with the highest risk were Lag4 and Lag06, with pooled *RRs* of 1.085(1.038, 1.134) and 1.468(1.244, 1.733). For SO_2_, the single-day lag and cumulative lag periods with the highest risk were Lag3 and Lag05, with pooled *RRs* of 1.036(1.013, 1.060) and 1.099(1.039, 1.161). For NO_2_, the single-day lag and cumulative lag periods with the highest risk were identified as Lag1 and Lag06, with pooled *RRs* of 1.121(1.069, 1.175) and 1.221(1.097, 1.359). For O_3_, the single-day lag and cumulative lag periods with the highest risk were Lag3 and Lag05, with pooled *RRs* of 1.084(1.052, 1.116) and 1.227(1.138, 1.323). Detailed results are presented in Figure 4.

## 4 Discussion

This study elucidates the cyclical patterns of emergency department visits for unintentional injuries in four cities in Shandong Province from 2019 to 2022 and systematically examines the association between air pollutants and emergency visits, including their lag effects. These results have helped to develop a general framework for the protection and control of unintentional injuries, while providing new evidence for the role of environmental factors in public health emergencies.

### 4.1 Cyclic characteristics of emergency department visits for unintentional injuries

The analysis revealed an annual cyclical pattern in emergency department visits for unintentional injuries across the four studied cities in Shandong Province, with distinct peaks concentrated in summer. In summer, rising temperatures increase the outdoor activities that residents participate in, which leads to a higher incidence of unintentional injuries such as drowning, high-temperature-related diseases, and traffic injuries. This cyclical nature suggests that public health institutions should develop seasonal interventions to prevent and control these injuries. For example, adding water safety management in summer will help reduce the risk of unintentional injuries.

### 4.2 Impact of air pollutants on emergency department visits for unintentional injuries

This study showed that increased concentrations of PM_2.5_ and PM_10_ were significantly associated with an increased risk of unintentional injuries, which is consistent with previous studies. A study conducted in South Korea found that there was a significant correlation between exposure to air pollution and unintentional injuries (8). Guo et al. also pointed out that based on the dose response, the risk of injury associated with falls is related to environmental PM_2.5_ (9). Heft-Neal et al. observed a nonlinear relationship between emergency department visits and smoke exposure, noting a 1–1.5% increase in total emergency visits within 1 week following low-to-moderate smoke days compared to smoke-free days (10).

The underlying mechanisms may involve two pathways. Firstly, particles (PM_2.5_ and PM_10_) can penetrate deep into the respiratory tract and lungs, leading to airway inflammation and mucosal overreaction (11). This reduces respiratory function by limiting the body’s oxygen supply and the elimination of carbon dioxide, which may lead to the disruption of physiological homeostasis, including a reduction in balance control and reaction time, and an increase in the risk of unintentional injuries. Secondly, elevated levels of PM_2.5_ and PM_10_ reduce atmospheric visibility (12), thus affecting people’s ability to clearly perceive the surrounding environment and related hazards when doing outdoor activities (such as walking or driving). The reduction in visual ability may further increase the risk of unintentional injuries, including car accidents, falls, and other injuries.

Research results show that when the concentrations of SO_2_, NO_2_, and CO increase, the risk of unintentional injury increases. These results are consistent with several previously reported studies. A study conducted in South Korea showed that all measured air pollutants were significantly associated with an increase in mortality from unintentional injuries, as well as emphasized the effects of inhalation of SO_2_, NO_2_, and CO on individuals aged≥1 year (13). Kampa et al. reported that air pollutants SO_2_, NO_2_, and CO have chronic and acute effects on human health (14). Petruzzi et al. showed that SO_2_ exposure can have short-term and long-term effects on the respiratory and other physiological systems, which may indirectly increase the risk of unintentional injuries (15). In addition, Pu et al. applied linear regression analysis of panel data to confirm the correlation between the increase in SO_2_, NO_2_, and CO levels and the increase in mortality caused by unintentional injuries (16). Based on potential mechanisms, there is some evidence that pollutants such as SO_2_, NO_2_, and CO may act as irritants to the respiratory mucosa, inducing inflammation, leading to airway contraction and decreased lung function (17). They may also increase blood viscosity and the risk of thrombosis (18). These physiological changes may also indicate potential risk factors for unintentional injuries.

The results also found a positive correlation between the increase in O_3_ concentrations and risk of unintentional injuries. Previous studies have shown that O_3_ exposure may lead to an increase in the incidence of depression (19). These psychological effects can lead to health-related anxiety, reduce attention, and impair awareness of environmental hazards and decision-making, thereby potentially increasing the risk of injury. Longitudinal studies also show that long-term exposure to air pollution is mainly associated with O_3_ and the decline of heart metabolic health in early adulthood (20), which may make individuals vulnerable to unintentional injury when engaged in daily activities. Emerging evidence shows that O_3_ exposure regulates immune function (21). In general, these pathophysiological changes can explain the “dangerous effect on the incidence of unintentional injuries.”

### 4.3 Lag effects of air pollutants on emergency visits for unintentional injuries

The research results show that all six types of air pollutants have significant single-day lag effects on emergency visits for unintentional injuries, with the risks for each air pollutant occurring at different lag periods: PM_2.5_ at Lag4, PM_10_ at Lag5, CO at Lag4, SO_2_ at Lag3, NO_2_ at Lag1, and O_3_ at Lag3. These lag differences may be due to different physical and chemical properties and biological mechanisms. For example, due to the irritation of SO_2_, it may induce acute respiratory irritation, resulting in impaired attention during exposure and ultimately increasing the risk of unintentional injuries. In contrast, particulate matter (PM_2.5_ and PM_10_), capable of entering systemic circulation via the respiratory tract, may require prolonged accumulation to exert health effects, resulting in more pronounced lag periods.

Cumulative lag analysis shows that the highest risks of PM_2.5_, PM_10_, CO, SO_2_, NO_2_, and O_3_ are between Lag05 and Lag07, indicating that both long-term exposure to low concentrations and short-term exposure to high concentrations may exacerbate the health effects leading to unintentional injuries. The most important cumulative effect was CO (*RR =* 1.468), which can be explained by its high toxicity. Continuous low exposure to carbon monoxide may damage cognitive function and reaction time, thus affecting the function of the central nervous system, eventually increasing the possibility of falls, traffic accidents or other unintentional injuries related to coordination disorders.

### 4.4 Study implications and limitations

The results of this research not only provide a scientific basis for the formulation of targeted and effective injury prevention plans, especially in Shandong Province, China, but also the impact of these findings can be used in similar areas with common climatic characteristics.

Several limitations should be acknowledged. Certain confounding factors (such as social behavioral factors and individual factors) were not included in stratified analyses, potentially leading to biased estimates of the association between air pollution and emergency department visits for unintentional injuries. Future research could include stratified analyses to further investigate the association between air pollutants and emergency department visits for unintentional injuries.

## Data Availability

The raw data supporting the conclusions of this article will be made available by the authors, without undue reservation.

## Footnotes

1
https://www.aqistudy.cn/

2
http://data.cma.cn/

## References

[1] Jullien S. Prevention of unintentional injuries in children under five years. BMC Pediatr. (2021) 21:311. doi: 10.1186/s12887-021-02517-2, 34496772 PMC8424785

[2] Haji Aghajani M Haddadi M Saadat S. Epidemiological pattern of injuries in Iran; a nationwide review of seven million emergency department admissions. Emergency (Tehran, Iran). (2017) 5:e10 28286817 PMC5325878

[3] de Ramirez SS Hyder AA Herbert HK Stevens K. Unintentional injuries: magnitude, prevention, and control. Annu Rev Public Health. (2012) 33:175–91. doi: 10.1146/annurev-publhealth-031811-124558, 22224893

[4] Ni T Chen M Zhou W Zhao J Jia D. Difference of achievements between physicians from public hospitals and emergency medical center in prehospital emergency. Medicine. (2018) 97:e13070. doi: 10.1097/md.0000000000013070, 30383688 PMC6221651

[5] Robertson LS Zhou L Chen K. Temperature, precipitation, ozone pollution, and daily fatal unintentional injuries in Jiangsu Province, China during 2015-2017. Inj Epidemiol. (2020) 7:42. doi: 10.1186/s40621-020-00268-9, 32713351 PMC7384214

[6] Fisher ZF Bollen KA Gates KM. A limited information estimator for dynamic factor models. Multivar Behav Res. (2019) 54:246–63. doi: 10.1080/00273171.2018.1519406, 30829065 PMC7473595

[7] Ma P Wang S Fan X Li T. The impacts of air temperature on accidental casualties in Beijing, China. Int J Environ Res Public Health. (2016) 13:1073. doi: 10.3390/ijerph13111073, 27827842 PMC5129283

[8] Jung J Kim G Kang SW Jeong S Kang Y Lee JY . Short-term exposure to ambient air pollution and injuries due to external causes according to intentions and mechanisms. Sci Total Environ. (2024) 912:169202. doi: 10.1016/j.scitotenv.2023.169202, 38097073

[9] Guo Y Lin H Shi Y Zheng Y Li X Xiao J . Long-term exposure to ambient PM(2.5) associated with fall-related injury in six low- and middle-income countries. Environ Pollution (Barking, Essex: 1987). (2018) 237:961–7. doi: 10.1016/j.envpol.2017.10.134., 29128246

[10] Heft-Neal S Gould CF Childs ML Kiang MV Nadeau KC Duggan M . Emergency department visits respond nonlinearly to wildfire smoke. Proc Natl Acad Sci USA. (2023) 120:e2302409120. doi: 10.1073/pnas.2302409120, 37722035 PMC10523589

[11] Abelenda-Alonso G Satorra P Marí-Dell'Olmo M Tebé C Padullés A Vergara A . Short-term exposure to ambient air pollution and antimicrobial use for acute respiratory symptoms. JAMA Netw Open. (2024) 7:e2432245. doi: 10.1001/jamanetworkopen.2024.32245, 39240563 PMC11380104

[12] Chen H Xu Y Gao Z Kang J Jiang Y Li Z . Visibility forecast in Jiangsu province based on the GCN-GRU model. Sci Rep. (2024) 14:12599. doi: 10.1038/s41598-024-61572-8, 38824165 PMC11144213

[13] Tager IB Balmes J Lurmann F Ngo L Alcorn S Künzli N. Chronic exposure to ambient ozone and lung function in young adults. Epidemiology. (2005) 16:751–9. doi: 10.1097/01.ede.0000183166.68809.b0., 16222164

[14] Kampa M Castanas E. Human health effects of air pollution. Environ Pollution (Barking, Essex: 1987). (2008) 151:362–7. doi: 10.1016/j.envpol.2007.06.012, 17646040

[15] Petruzzi S Musi B Bignami G. Acute and chronic Sulphur dioxide (SO2) exposure: an overview of its effects on humans and laboratory animals. Ann Ist Super Sanita. (1994) 30:151–6. 7832407

[16] Pu H Li B Luo D Wang S Wang Z Zhao W . Impact of urbanization factors on mortality due to unintentional injuries using panel data regression model and spatial-temporal analysis. Environ Sci Pollut Res Int. (2020) 27:2945–54. doi: 10.1007/s11356-019-07128-0, 31838677

[17] Han C Jang M Yoon J Lee B Kim J Jang H . Estimating the acute health effects of smoke exposure from an urban factory fire accident: a case study of a Tire factory fire in Korea. Environ Health Perspect. (2024) 132:87008. doi: 10.1289/ehp14115, 39196399 PMC11353213

[18] Borsi SH Khanjani N Nejad HY Riahi A Sekhavatpour Z Raji H . Air pollution and hospital admissions due to deep vein thrombosis (DVT) in Ahvaz, Iran. Heliyon. (2020) 6:e04814. doi: 10.1016/j.heliyon.2020.e04814. 32913913 PMC7472851

[19] Cao T Tian M Hu H Wu H Yu Q Su X . Do social economic status modify the association between air pollution and depressive or anxiety symptoms? A big sample cross-sectional study from the rural areas of Central China. J Affect Disord. (2024) 362:502–9. doi: 10.1016/j.jad.2024.07.063, 39025437

[20] Zhu L Fang J Yao Y Yang Z Wu J Ma Z . Long-term ambient ozone exposure and incident cardiovascular diseases: national cohort evidence in China. J Hazard Mater. (2024) 471:134158. doi: 10.1016/j.jhazmat.2024.134158, 38636234

[21] Jakab GJ Spannhake EW Canning BJ Kleeberger SR Gilmour MI. The effects of ozone on immune function. Environ Health Perspect. (1995) 103:77–89. 10.1289/ehp.95103s277 PMC1518840 7614952

